# Stiffness in breast masses with posterior acoustic shadowing: significance of ultrasound real time shear wave elastography

**DOI:** 10.1186/s12880-022-00797-3

**Published:** 2022-04-17

**Authors:** Hui Luo, Jian Li, Yang Shi, Xiaojun Xiao, Yuanyang Wang, Zhanghong Wei, Jinfeng Xu

**Affiliations:** 1 Department of Ultrasound, Shenzhen Medical Ultrasond Engineering Center, Shenzhen People’s Hospital, The Second Clinical Medical College of Jinan University, The First Affiliated Hospital of Southern University of Science and Technology, Shenzhen, 518020 Guangdong China; 2 Thyroid and Breast Surgical Department of Shenzhen People’s Hospital, The Second Clinical Medical College of Jinan University, The First Affiliated Hospital of Southern University of Science and Technology, Shenzhen, 518020 Guangdong China

**Keywords:** Ultrasound, Real time shear wave elastography, Breast masses, Posterior acoustic shadowing, Stiffness

## Abstract

**Background:**

To assess the stiffness of benign breast masses in ultrasound images with posterior acoustic shadowing (PAS) and malignant lesions, and explore the significance of differential diagnosis using ultrasound real time shear wave elastography.

**Material and methods:**

All 117 mammary masses (98 patients) with PAS were assessed by using routine ultrasound examination, and elastic modulus values were obtained with the real time shear wave elastography mode. All breast lesions were confirmed by surgery or biopsy. The significance of differences in ultrasound elastography values between breast benign and malignant masses with posterior acoustic shadowing was assessed, and the ROC curves of elasticity modulus values were analyzed.

**Results:**

Among the 117 masses, 72 were benign and 45 were malignant. The two types of breast masses showed significant differences in size, margin, internal echo, calcification, and blood flow characteristics (*P* < 0.05), although the difference in orientation was not significant (*P* > 0.05). *Emean*, *Emax* and *Esd* obtained with real time shear wave elastography showed statistically significant differences between benign masses with posterior acoustic shadowing and breast cancer (*P* < 0.05), while *Emin* showed no significant difference between them (*P* = 0.633). Ultrasound real time shear wave elastography showed higher sensitivity and specificity than conventional ultrasound.

**Conclusions:**

Benign and malignant breast masses with PAS show different ultrasound manifestations. Real time shear wave elastography can facilitate the differential diagnosis and treatment planning for these breast masses.

## Introduction

In the dictionary of Breast Imaging Reporting and Data System (BIRADS), posterior acoustic shadowing (PAS) is a term used to describe an ultrasound characteristic that appears in many breast masses. Masses with acoustic shadowing are usually judged as category 4 lesions in BIRADS by conventional ultrasound. However, many masses with acoustic shadowing turn out to be benign lesions in postoperative pathological assessments. The posterior acoustic shadowing has been reported to be a feature of both malignant and benign breast lesions [1, 2], some researchers have observed that not only breast cancer but also breast fibrocystic lesions, fat necrosis, and postoperative scars could be with acoustic shadowing. How to distinguish between benign masses with PAS and malignant masses? It is well known that benign masses are soft and malignant masses are hard. Can hardness information be used to differentiate breast masses with acoustic shadowing? E imaging can reveal the hardness of masses. Real Time Shear Wave Elastography (RTSWE) for short as "E imaging", it including Sound Touch Elastography (STE) and strain elastography. This study aimed to explore the different ultrasound characteristics of benign and malignant breast masses with PAS, and use E imaging to explore the differences between the elasticity of benign masses with PAS and breast cancer. Our aim is to use these hardness assessments to distinguish benign masses with PAS and malignant lesions, thereby improve the accuracy of ultrasound diagnosis[3, 4].

## Materials and methods

### Patient selection

A total of 117 breast masses (98 patients) with PAS were selected from 635 patients who underwent ultrasound examinations at our hospital between January 2018 and October 2020. All patients were female. Ultrasound evaluated breast lesions using the BIRADS category. For all findings assessed as category 3 at ultrasound, were recommend Short-interval (6-month) follow-up or continued surveillance. When the masses became large or irregular during follow-up, a needle biopsy were recommended. All breast masses were confirmed by surgery or biopsy after E imaging examination, and obtained pathological examination results. The patients were aged 25–68 years (mean age, 45 ± 11 years), and the nodule size was 0.4–3.8 cm. Case inclusion criteria were as follows: (1) breast masses showing partial or complete PAS in sonography; (2) nodule diameter ≤ 4.0 cm; (3) completed follow-up assessments of the masses. We excluded cases that met the following criteria: (1) masses were open breast lesions, such as skin rupture, pus, or infection; (2) no intervention or surgery was performed for the masses before the ultrasound scans; and (3) the diameter of the masses was greater than 4.0 cm. The study was done after agreement from the local ethics committee and with the patients’ informed consent.

### Image acquisition and interpretation

All images were collected by physicians(YS, XX) with over 15 years of ultrasound diagnosis and 3 years of experience in E imaging at Shenzhen People’s Hospital, Guangdong, using a double-blind approach. Adopt Mindray Resona 7s ultrasonic diagnostic equipment equipped with the E imaging function and a 14–5 MHz linear array probe was used at a frequency of 5 ~ 14 MHz. The patients were examined in the supine position and underwent routine assessments for recording the lesion position, size, orientation, shape, boundary, internal echo, presence or absence of calcification, posterior echo, blood flow characteristics of the masses, and BIRADS category. The mass was entered completely into the region of interest (ROI) box, and elastic images of the mass was obtained and stored on the instrument. The stored images were then post-processed on the instrument to measure the maximum of the elastic modulus *(Emax*), average of the elastic modulus (*Emean*), minimum of the elastic modulus (*Emin*), and standard deviation of the elastic modulus (*Esd*).

### Statistical analysis

Statistical software SPSS22.0 was used for statistical analysis. The gold standard of diagnosis was the result obtained with pathological assessment after surgery or hollow needle biopsy (14G). The two groups were compared by using the t test for the mean of the two samples, and the counting data were assessed using the chi-squared test to compare whether the differences between ultrasonic characteristics and elastic imaging modulus values of benign breast masses with PAS and malignant lesions were statistically significant. The receiver operating characteristic (ROC) curve was used to analyze the elasticity modulus, and the optimal cut-off elasticity modulus value for differential diagnosis of benign breast masses with PAS and malignant lesions was obtained. *P* < 0.05 was considered statistically significant.

## Results

### Pathological outcome

A total of 117 breast masses were examined, including 72 benign lesions and 45 malignant lesions. Most benign lesions with PAS in the study were cases of fibrocystic mastopathy, which accounted for 67% (48/72) of benign breast masses, and cysts with eggshell calcifications, accounting for 15% (11/72). In addition, the masses included 6 cases of sclerotic adenopathy, 4 cases of radial scars, and 3 cases of granulomatous inflammation. The malignant lesions with PAS included 29 cases of invasive ductal carcinoma, accounting for 64% (29/45) of the breast malignant lesions, 10 cases of ductal carcinoma in situ, 2 cases of malignant phyllodes tumor, 3 cases of mucinous carcinomas, and 1 case of invasive lobular carcinoma.

### Ultrasound performance

**Fig. 1:**
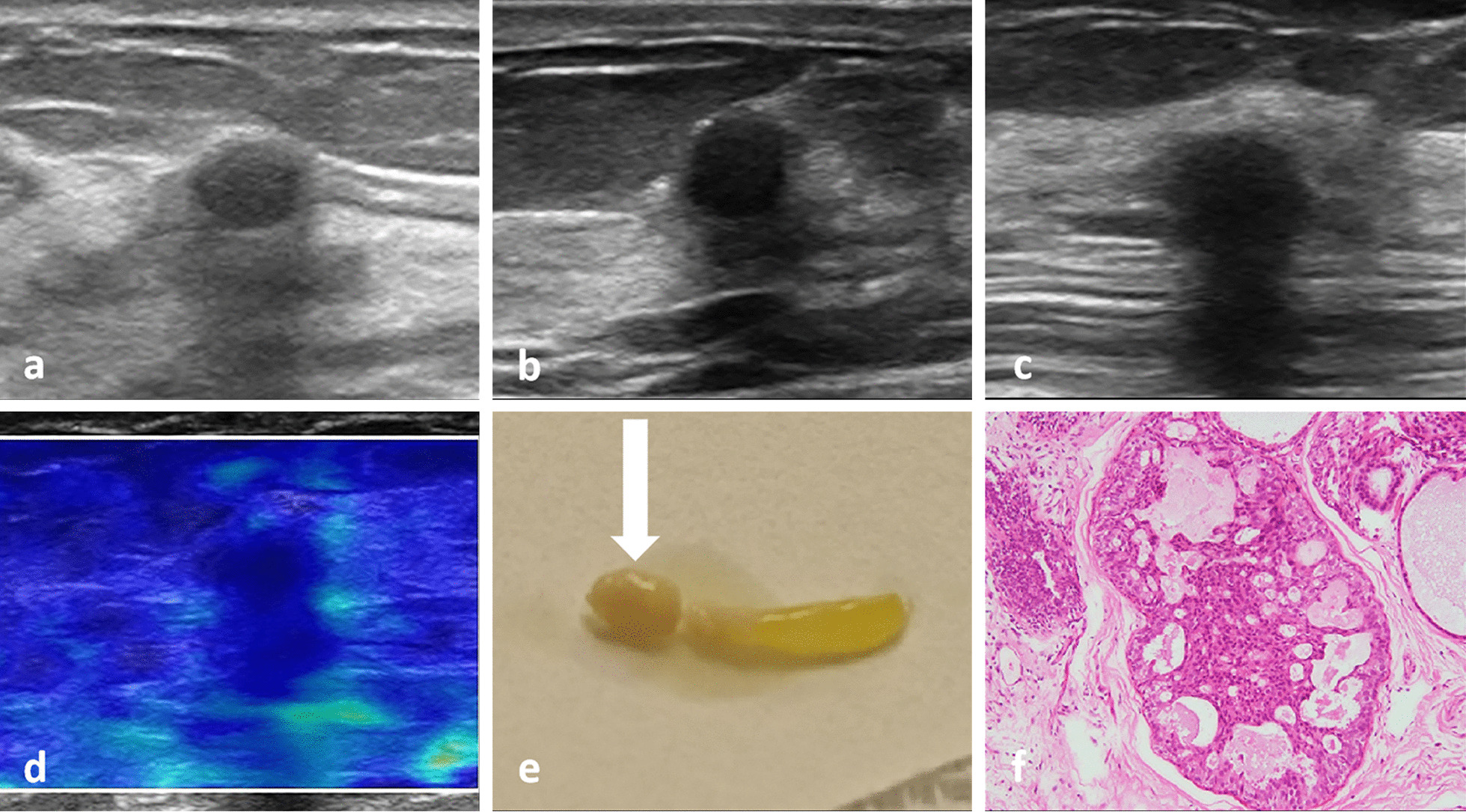
**a** 45-year-old female patient showing changes of posterior acoustic shadowing of a mammary nodule of fibrocystic mastopathy over time. In the first year, breast nodule image showing a weak posterior acoustic shadowing, oval shape, parallel position, and circumscribed margin (**a**). Over time, the nodule showed a non-parallel position (taller than wide or vertical), and the posterior acoustic shadowing was obvious (**b**). Subsequently, the posterior echo was significantly attenuated, and the posterior field was unclear (**c**). In E imaging of nodules, the elasticity of nodules was similar to that of the surrounding tissues. The elastic modulus value of the nodule was measured by E imaging, and Emax was 36 kPa (**d**). Powdery object was extractioned by hollow needle puncture (arrow) (**e**). The pathology results confirmed that the lesion indicated fibrocystic mastopathy (HE, 40 ×) **(f**)

**Fig. 2:**
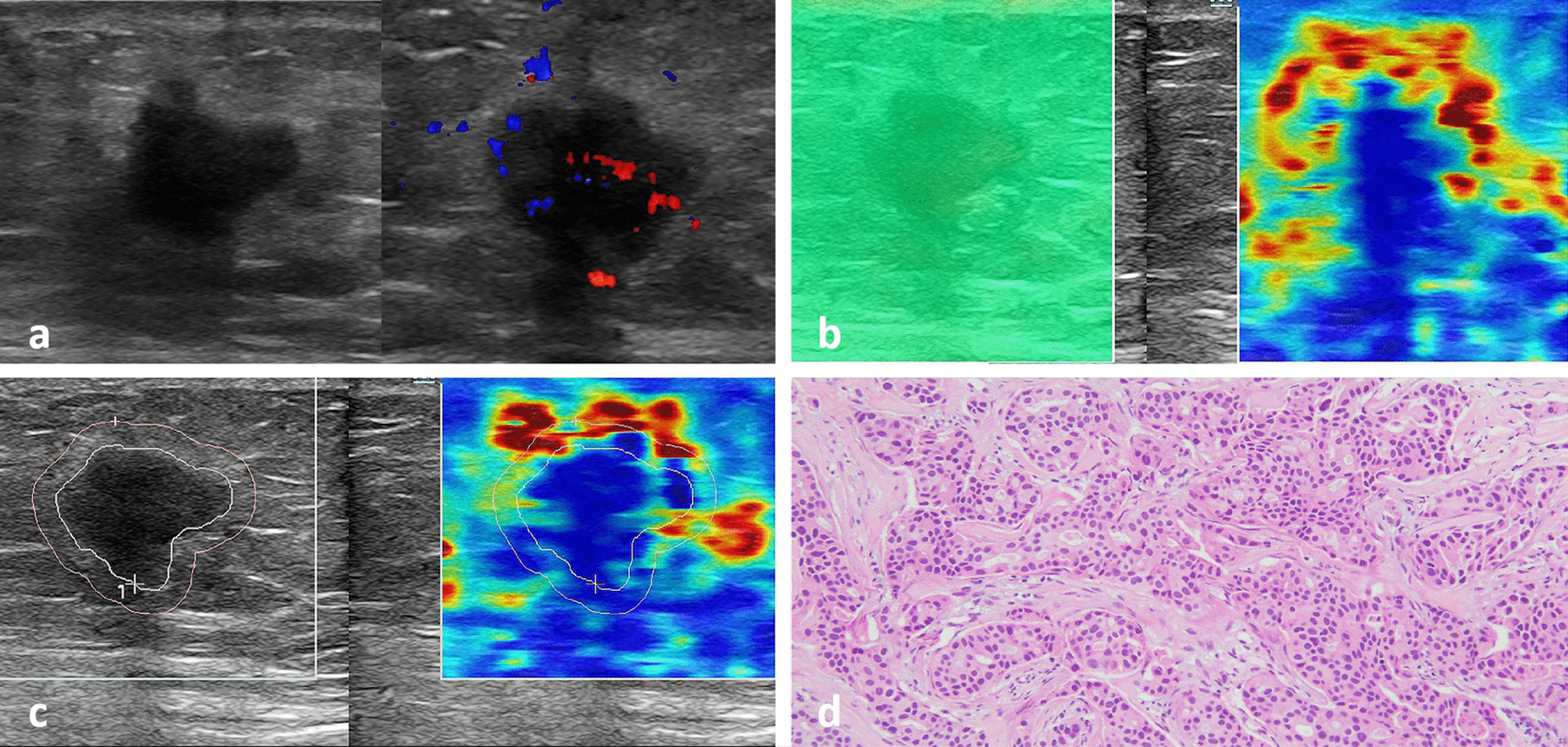
A 55-year-old female patient with an invasive non-special type breast cancer. Two-dimensional grayscale image of breast cancer showing posterior echo attenuation and rich blood flow signal inside the breast cancer (**a**). The probe was kept stable still before elastic imaging scanning, when the ROI area was almost all green, the image stability was good, and the elastic modulus value of the mass was measured with STE (**b**). E images showed that the mass was hard with a “stiff-rim” sign, the elastic modulus value of the nodule was measured by E imaging, and Emax was 133 kPa (**c**). The pathology results confirmed that the lesion was an invasive non-special type carcinoma (HE, 100 ×) (**d**)

During the follow-up observation, the posterior echo of some fibrocystic mastopathy masses changed from a weak acoustic shadowing to an obvious acoustic shadowing, and the shape gradually changed from a parallel to non-parallel position, after which the posterior field became unclear (Fig. 1). 59 cases were biopsied by hollow needle puncture, pathological displayed mammary gland disease, focal cyst. Breast cancer with acoustic attenuation has irregular morphology and rich blood flow signals inside (Fig. 2). In this study, the benign and malignant breast masses with PAS showed statistically significant differences in size, margin, internal echo, calcification, and blood flow characteristics (*P* < 0.05). There was no statistically significant difference in orientation (*P* > 0.05) (Table 1). The average diameter of benign masses was 0.4–1.5 (0.9 ± 0.5) cm; the average diameter of malignant masses was 0.7–3.8 (1.5 ± 0.5) cm (Table 2). The sensitivity of conventional ultrasound the diagnosis breast masses with PAS was 65%, and specificity was 68%.

**Table 1:** Comparison of ultrasound characteristics of 117 breast masses with PAS (cases)

	Benign N = 72	Malignant N = 45	χ^2^	*P*
Orientation			0.498	0.480
Parallel	24	19		
Non-parallel	48	26		
Margin			29.915	< 0.001
Circumscribed	52	5		
Not circumscribed	20	40		
Echo			12.955	< 0.001
Homogeneous	36	4		
Heterogeneous	36	41		
Calcification			8.353	0.004
Present	53	20		
Absent	19	25		
Vascularity			12.747	< 0.001
Present	45	11		
Absent	27	34

**Table 2:** Comparison of size and Elastography values ($\overline{x} \pm s$) of breast masses with PAS

Lesions	Size (cm)	Emax (kpa)	Emean (kpa)	Emin (kpa)	Esd
Benign (72)	0.9 ± 0.5	68 ± 31	32 ± 12	12 ± 6.5	13 ± 7.5
Malignant (45)	1.5 ± 0.5	125 ± 42*	44 ± 7†	13 ± 7.5‡	20 ± 9.2§
t	− 5.972	− 4.574	− 3.777	− 0.482	− 3.374
*P*	< 0.001	< 0.001	0.001	0.633	0.001

Compared with the benign group (t test).

^‡^*P* > 0.05, *Emin* was not significantly different. The other *P* values were less than 0.05. The average diameter, *Emax*, *Emean*, and *Esd* all showed significant differences

### Comparison of ultrasound elastography of benign breast masses with PAS and malignant breast masses

**Fig. 3:**
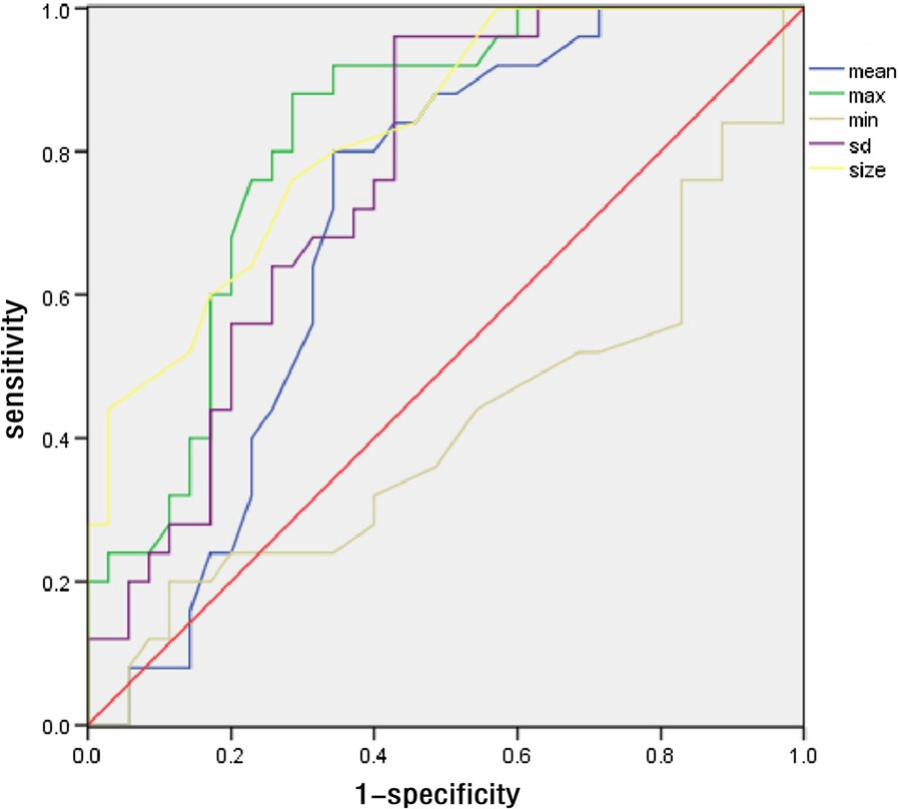
ROC curve analysis of elastic modulus value and size

**Fig. 4:**
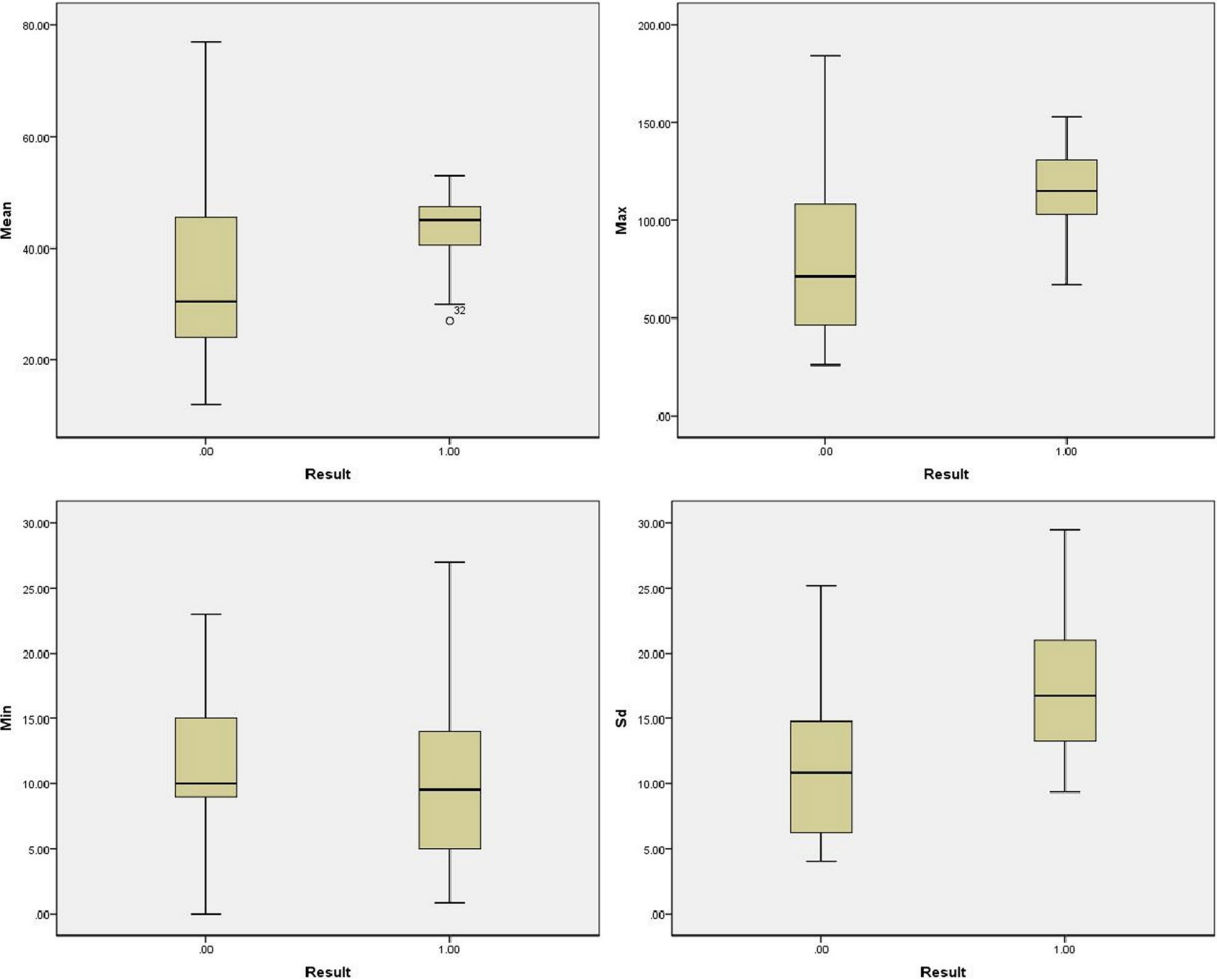
Boxplot of real time shear wave elastography of benign and malignant lesions. The 0.00 on the abscissa represented benign lesions and the 1.00 represented malignant masses. Emean, Emax and Esd of malignant masses were higher than those of benign lesions, and Emax was the most significant. Emin of benign lesions was higher than that of malignant masses

In this study, the elastic modulus values of benign lesions were smaller than those of malignant lesions; all 72 benign lesions displayed colors similar to those of surrounding tissues with either green or blue regions (Fig. 1), while the 45 malignant masses displayed a reddish periphery (red rim) and a blue region in the middle (Fig. 2). Using the *t* test for comparison of the mean values of the two samples, the differences in the *Emean*, *Emax, Esd* of benign breast masses with PAS and malignant masses were statistically significant (*P* < 0.05). There were no significant differences in *Emin* between two types of breast masses with PAS (*P* = 0.633) (Table 2). The AUC values of the ROC curve for the *Emax*, *Emean*, and *Esd* for the diagnosis of benign breast lesions with PAS and malignant lesions were 0.818, 0.701, and 0.762 (Fig. 3). The diagnostic efficiency of each elastic modulus value is shown in Table 3. *Emax* showed the highest sensitivity and specificity for the diagnosis of breast tumors with acoustic shadowing (Fig. 4). The sensitivity and specificity of Ultrasound E imaging diagnosis of breast masses with PAS were 88% and 72% respectively.

**Table 3:** Diagnostic thresholds and diagnostic efficacy of parenchyma elastic modulus values for benign and malignant breast masses with PAS

Modulus of elasticity	Cutoff (kPa)	AUC	Sensitivity (%)	Specificity (%)
Emax	95	0.818	88	72
Emean	31	0.701	88	51
Esd	12	0.762	96	53

AUC = area under the receiver operating characteristic curve

## Discussion

When a sound wave propagates in a medium, it loses its energy due to reflection, refraction, scattering, and other effects. This phenomenon of reduced sound energy during propagation is called sound attenuation, and can be used to visualize stones, calcification, and bones [5]. Ultrasound attenuation is closely related to tissue density and composition. In the literature, the posterior attenuation of malignant breast lesions is due to collagen and collagen fibers within the tumor tissue, necrotic bleeding, and calcification, resulting in ultrasound energy attenuation. Of the 45 breast cancers with PAS in this study, 25 showed calcifications. The higher the degree of tissue degeneration and necrosis, the more obvious the attenuation of the mass [6]. However, current ultrasound image segmentation classification technology cannot solve the problems associated with PAS. Zhou et al. observed that “the upper half of the tumor contour is less affected by PAS” [1], who proposed that “half-contour features were proposed to classify breast tumors with PAS”. Some image processing experts have made some useful explorations in improving image quality. On the other hand, sonographers can use elastography to overcome the limitations of acoustic attenuation and more accurately diagnose breast tumors with acoustic shadowing [7, 8].

This study included 13 cysts with eggshell calcifications and obvious acoustic shadowings. Of the 48 cases of fibrocystic mastopathy with PAS, 46 involved women with a history of pregnancy and lactation. Sixteen of these patients had 2 masses with PAS, one had three masses with PAS, and 29 had concurrent multiple small cysts. The common characteristics of most masses of fibrocystic mastopathy were a smooth surface, circumscribed margin, round or oval shape, hypoechoic, homogeneous echo, no vascularity. During the course of the three-year follow-up visit, the echo characteristics of 20 accompanying small cysts changed: the echo became lower; the posterior acoustic shadowing changed from weak to obvious; the orientation changed from parallel to non-parallel; and the margin of the back field was unclear. The ultrasound appearance became similar to that of the fibrocystic mastopathy masses. Some researchers [2, 9, 10] have reported the appearance of PAS on ultrasound sonograms in cases of fibrocystic mastopathy, diabetic breast disease, fat necrosis, postoperative scars, focal fibrosis, sclerosing adenopathy, and other diseases, which pathologically manifested as a mass of collagen and spindle-like stromal cells and fibrous elastic tissue hyperplasia. In comparison with reports in the literature, benign cases in this study group only showed fibrocystic mastopathy, cysts with eggshell calcifications, sclerosing adenopathy, scars, granulomatous inflammation with acoustic shadowing.

This study also showed that the growth orientation of both benign and malignant masses with PAS is mainly non-parallel, with 48 of 72 benign masses (67%) and 26 of 45 malignant masses (58%) being non-parallel. The proportion of non-parallel benign masses with PAS was more than that of malignant masses, which is different from the findings for benign masses without PAS. Multiple reports in the literature have described the orientation of breast masses as one of the indicators to identify benign and malignant masses, benign masses were mainly parallel and malignant masses were mainly non-parallel. Kim et al. [11]. studied 34 breast fibroadenomas and found that 32 were parallel while 2 were non-parallel; Choi et al. [12]. and other authors also pointed out that non-parallel positioning was an independent factor for malignant tumors, and its sensitivity, specificity, and AUC for breast cancer diagnosis were 84.2%, 83.9%, and 0.90, respectively. Nouri-Neuville et al. [13]. reported that the benign predictive value of parallel orientation for masses was greater than 0.85. In this study, most benign masses with PAS were non-parallel, which was different from the findings for benign masses without PAS. This discrepancy may be attributed to the fact that the benign masses with PAS in this study mostly involved fibrocystic mastopathy (67% of benign masses), and PAS formation in fibrocystic mastopathy was attributable to thickening of the cystic fluid, decreased water in the cyst, concentration of cyst ingredients, and gradual desiccation and hardening followed by solidification and contraction to form a round shape. These circular nodules showed a non-parallel position and small size on ultrasound. Most benign masses with PAS in this study were less than 1 cm in size, with an average diameter of 0.9 ± 0.5 cm; the malignant masses with PAS were unequal in size, with an average diameter of 1.5 ± 0.5 cm, and the sizes of the benign and malignant masses were statistically different.

The fibrocystic mastopathy and cysts with eggshell calcifications mainly contained secretions and exfoliated cells, so the ultrasound images showed homogeneous echo, circumscribed margin, posterior acoustic shadowing, and a slightly soft texture. In contrast, inflammatory granulomas, sclerosing adenopathy, and radial scars had a fibrous appearance with connective tissue hyperplasia, which may be accompanied by liquefaction, so their echo was heterogeneous and they showed not circumscribed margin, irregular shape, posterior combined pattern, and a slightly hard texture. Breast cancers showed an irregular shape, heterogeneous echo, degenerative necrotic cells and cell debris, calcifications, and posterior combined pattern. The tumor tissue stimulated the surrounding fibrous connective tissue, which led to collagenization of the hyperplastic fibrous tissue with a rich interstitial composition, so the mass was hard and showed large hardness values on elastography. The common point was that these masses contained collagen, which was responsible for the posterior attenuation [14, 15]. The ultrasound images of the two groups were different: the benign masses with PAS were mostly uniform in composition, with homogeneous echo, circumscribed margin and posterior acoustic shadowing, and only 19 of the 72 masses were calcified, of which 11 showed ring-shaped or curved calcification, while color Doppler flow imaging mostly showed no internal blood flow signal. In contrast, 25 of 45 breast cancers showed calcification embedded in masses with heterogeneous echo and posterior combined pattern; 40 cancers were not circumscribed margin, while color Doppler blood flow imaging showed a rich blood flow signal inside 34 masses. The ultrasound characteristics of benign masses reported in previous studies were consistent. For example, in 34 breast fibroadenomas reported by Kim [11], included 22 masses (65%) with posterior enhancements, 1 with PAS, 9 without posterior acoustic features; 32 with circumscribed margins, 2 with halos, 3 with calcifications, and 31 without calcifications. The use of STE to visualize the hardness of the mass helped differentiation between benign masses and malignant lesions with PAS. The tissue composition of granulomatous inflammation was complex, with sclerosing adenopathy and radial scars, and the elasticity values were between those of fibrocystic mastopathy and breast cancer.

Benign masses are soft, and malignant masses are hard in texture. Benign masses are less stiff than malignant masses. Ultrasound elastography can distinguish benign and malignant masses by characterizing tissue hardness, and is especially suitable for studies of superficial tissues and organs [16, 17]. The basic principle of STE imaging is that a small strain is applied to the biological tissue through the mechanical action of external forces, and then the deformation degree of the tissue is monitored by ultrasound, so as to calculate the offset, strain or elastic modulus of the tissue. Therefore, the STE imaging is also called as “ultrasonic elastic imaging”. STE calculates the elastic modulus of the tissue at the detected shear wave propagation speed [18], in kPa, and color-codes the image at the same time to obtain color shear-wave images. In this study, benign masses with PAS mainly showed blue color on elasticity imaging, which was consistent with the color of surrounding tissues, and the *Emean*, *Emax*, and *Esd* values were small. In contrast, elasticity imaging of breast cancer with PAS usually showed a blue color and a surrounding ring-shaped or semi-ring-shaped red rim, and after the outline of the tumor was delineated on the conventional two-dimensional grayscale image, the red hard ring of the elastic image extended beyond the contour, and the nodule showed large *Emean*, *Emax*, and *Esd* values. The difference between the *Emean*, Emax, and Esd values of benign masses with PAS and malignant masses was statistically significant (*P* < 0.05), indicating that E imaging was an effective method for differential diagnosis of breast masses with PAS [19–22]. The results of this study were consistent with those reported by Zhou et al. [23], who called the color distribution of the peripheral red and inner blue of breast cancer in shear-wave elastic imaging as the “stiff-rim” sign and pointed out that increased peripheral stiffness was a specific manifestation of breast cancer(red depicted as hard and blue as soft). They proposed that the “stiff-rim” sign could be used to diagnose breast cancer with an AUC of 0.918, and that the formation of the “stiff-rim” sign may be related to tumor invasion. Studies have shown that breast cancer is often accompanied by a large amount of fibrous tissue hyperplasia and cross-linking, and a large number of expanded cancer cells form cancer nests, causing hardening of the extracellular matrix and the formation of a ring of hard fibrous tissue around the tumor. The presence of thick collagen fibers in the tumor boundary was related to the aggressiveness of breast cancer. With an increase in collagen, the hardness increases, and the breast cancer becomes more invasive [24, 25].

The study showed that PAS can appear in both benign and malignant breast masses. The differential diagnosis of these masses could be preliminarily based on the ultrasound findings for size, circumscribed margins, internal echo, presence or absence of calcification, and blood flow characteristics. The orientation did not influence the differential diagnosis of masses with PAS. Thus, more frequent use of E imaging to measure the hardness of masses could improve the accuracy of diagnosis and ensure correct identification of benign and malignant breast masses with PAS, which is of great significance in improving the quality of life of patients.

The present study has some limitations. First, the sample size is small, especially the number of cases of sclerosing adenopathy, scar and granulomatous inflammation with PAS is small; secondly, the elastic modulus values measured by different instruments are different, and the elastic values between different instruments are not compared. To expand the sample size, we will carry out further research.

In conclusion, benign breast masses with PAS differ in orientation from masses without PAS. E imaging may reflect the hardness of breast lesions with PAS. It is an effective differential examination method and can be used for the diagnosis of breast masses.

## Data Availability

The datasets during and/or analyzed during the current study are available from the corresponding author on reasonable request.

## Abbreviations

BIRADS: Breast Imaging Reporting and Data System
RTSWE (E imaging): Real Time Shear Wave Elastography
ROI: Region of Interest
Emean: Average of the elastic modulus
Esd: Standard deviation of the elastic modulus
PAS: Posterior Acoustic Shadowing
STE: Sound Touch Elastography
Emax: Maximum of the elastic modulus
Emin: Minimum of the elastic modulus
ROC: Receiver operating characteristic curve
